# Extreme Rhabdomyolysis Secondary to Influenza A Infection

**DOI:** 10.7759/cureus.101013

**Published:** 2026-01-07

**Authors:** Ricardo Velho, Manuel Maia, Bernardo Belchior, Leandro Valente, João Coelho, José Brito, Joana Marinho Silva, Diogo Leal, João Miguel Peixoto, Ana Sofia Teixeira

**Affiliations:** 1 Internal Medicine, Unidade Local de Saúde de Coimbra, Coimbra, PRT; 2 Internal Medicine, Centro Hospitalar e Universitário de Coimbra, Coimbra, PRT; 3 Infectious Diseases, Unidade Local de Saúde de Coimbra, Coimbra, PRT

**Keywords:** acute kidney injury, creatine kinase, influenza a, muscle tissue, rhabdomyolysis

## Abstract

Rhabdomyolysis is a clinical syndrome characterized by the breakdown of skeletal muscle cells, leading to the release of intracellular contents into the bloodstream. If left untreated, this disease can cause serious complications, such as myoglobinuric acute kidney injury, electrolyte imbalances, arrhythmias, and death. Prompt diagnosis and treatment of rhabdomyolysis is fundamental to avoid progression to severe complications. It can be caused by trauma, strenuous exercise, drugs, and infections. The authors present a case of a previously healthy 33-year-old male who presented to the emergency department with flu-like symptoms and generalized muscle pain. Extreme rhabdomyolysis was detected, with abnormally high serum creatine kinase levels. Despite the extreme elevation of creatine kinase, the patient did not develop acute kidney injury. Other common and rare causes of rhabdomyolysis were excluded. Although influenza A infections do not commonly cause severe rhabdomyolysis, this case highlights the importance of considering this diagnosis in patients with influenza-like symptoms and prompt diagnosis and management to prevent serious complications.

## Introduction

Rhabdomyolysis is a syndrome characterized by the disintegration and necrosis of muscle cells and the release of their intracellular contents (mainly creatine kinase (CK), myoglobin, and potassium) to the bloodstream. The hallmark of this syndrome is the elevation of CK levels, although other serum muscle enzymes are also elevated [1]. Clinically, patients with rhabdomyolysis present with muscle pain, fatigue, and dark-colored urine (myoglobinuria) [2]. This syndrome is potentially life-threatening due to the risks of myoglobinuric kidney injury, electrolyte imbalances, and cardiac arrhythmias. Rhabdomyolysis can have multiple causes, the most recognizable ones being trauma, strenuous exercise, drugs, and metabolic disorders [3]. Infectious diseases can also cause rhabdomyolysis, including viral, bacterial, fungal, and protozoal infections. Among viral agents, influenza A is a virus that can cause rhabdomyolysis due to mechanisms related to direct viral invasion of muscle tissue, immune-mediated muscle damage, and systemic inflammatory response. Direct invasion of myocytes by the virus and the release of proinflammatory cytokines lead to myonecrosis and the development of muscle tissue damage [4]. Although rhabdomyolysis associated with influenza A infection is a recognized complication, reported cases usually describe mild to moderate CK elevation and occur predominantly in pediatric or immunocompromised patients [5]. Reports of extreme rhabdomyolysis in previously healthy adults are scarce, and the absence of acute kidney injury in such severe presentations is rare. We present this case to highlight the broad clinical spectrum of rhabdomyolysis associated with influenza A infection and to emphasize the importance of early recognition and aggressive support management to prevent renal complications. 

## Case presentation

Our report describes a 33-year-old male with no past medical history or regular medication who presented to the emergency department in January 2024 with a 48-hour history of cough, fever, sore throat, and myalgias. The physical examination showed no abnormal features, including the absence of respiratory distress signs, and he had no fever on admission. The patient tested positive for influenza A virus infection using reverse transcriptase polymerase chain reaction tests. Laboratory workup showed an extreme elevation of CK levels (59407 U/L), as well as elevated lactate dehydrogenase levels (1039 U/L), alanine aminotransferase levels (340 U/L), and aspartate aminotransferase levels (1276 U/L). Myoglobin levels on admission were 8863 ng/mL. Other blood tests on admission were normal, including normal renal function and normal potassium levels. Further investigation showed that the patient had no history of trauma, physical exertion, or drug abuse; he also had no history of any known genetic or metabolic disease. Additional laboratory tests were done. Urinalysis showed turbid urine, and urine microscopy had no abnormal findings. Urine toxicology screening was negative for cocaine, cannabinoids, benzodiazepines, amphetamines, and alcohol. Other viral infections were screened, including coronavirus, influenza B, human immunodeficiency virus, hepatitis A, B, and C, Epstein-Barr virus, cytomegalovirus, and coxsackievirus, all of which were negative. Chest radiography and abdominal ultrasound showed no significant findings.

On admission, as soon as the first laboratory results became available, aggressive intravenous fluid therapy with normal saline solution (0.9% NaCl) was initiated, using an initial fluid rate of 500 mL per hour for the first six hours. During hospitalization, increased oral hydration, intravenous fluids, and bed rest led to a progressive symptom improvement (which disappeared completely after two days) and normalization of CK levels. After five days, CK levels decreased to 2159 U/L, as seen in Table 1. 

**Table 1:** Abnormal serum laboratory results during hospitalization and their evolution U = units; L = Liter

Serum parameter	Day 1	Day 2	Day 3	Day 4	Day 5	1 month	Range
Creatine kinase (U/L)	59407	37213	20728	12183	2159	152	< 171
Lactate dehydrogenase (U/L)	1039	926	859	842	500	192	< 248
Alanine aminotransferase (U/L)	340	335	240	210	169	25	< 45
Aspartate aminotransferase (U/L)	1276	1137	944	661	129	32	< 35

The patient was discharged after five days of hospitalization with marked biochemical improvement and complete resolution of symptoms. He remained under outpatient follow-up, with repeat laboratory evaluation one month after discharge demonstrating normalization of CK and liver enzyme levels, as well as preserved renal function. During long-term follow-up over the subsequent years, the patient remained asymptomatic and experienced no new episodes of rhabdomyolysis.

## Discussion

Rhabdomyolysis is a recognized complication of viral infections. Influenza A virus can cause rhabdomyolysis, although, in most reported cases, it is characterized by mild to moderate elevations in CK levels and predominantly affects pediatric patients or individuals with underlying comorbidities, such as immunosuppression or chronic systemic disease [1,5-7]. However, severe or extreme rhabdomyolysis in previously healthy adults remains rare and is likely underreported, making this case clinically relevant.

The degree of muscle injury observed in this patient was markedly higher than that typically described in literature, with CK levels exceeding 59,000 U/L. The extreme biochemical severity in this case, occurring in the absence of trauma, intense physical exertion, substance use, or metabolic disease, strongly suggests influenza A infection as the primary etiological factor. Proposed mechanisms include direct viral invasion of miocytes, immune-mediated muscle damage, and cytokine-driven systemic inflammation, all of which may contribute to myonecrosis and muscle cell membrane disruption [5,6]. The exact reason why some immunocompetent adults develop such severe muscle injury remains unclear but may be related to host-specific immune responses or viral strain virulence [5].

Despite the extreme elevation of CK levels, this patient did not develop acute kidney injury, which represents an atypical and clinically significant finding. Acute kidney injury is one of the most feared complications of rhabdomyolysis and is primarily mediated by renal vasoconstriction, tubular obstruction by myoglobin casts, and direct tubular toxicity by myoglobin [3,8,9]. In this case, early recognition of rhabdomyolysis and prompt initiation of intravenous fluid therapy likely played a crucial role in preserving renal function, highlighting the importance of timely management even in patients with preserved renal parameters at presentation.

Another relevant aspect of this case is the marked elevation of liver enzymes, particularly aspartate aminotransferase, in the absence of intrinsic liver disease. This pattern has been previously described in severe rhabdomyolysis and reflects the concentration of aminotransferases within skeletal muscle cells rather than true hepatic injury [10-12]. Failure to recognize this phenomenon may lead to unnecessary hepatological investigations and misinterpretation of disease severity. The parallel decline of aminotransferases and CK levels during recovery further supports a muscular origin of these abnormalities.

This case reinforces the need for heightened clinical suspicion of rhabdomyolysis in patients presenting with influenza-like symptoms and prominent myalgia. Early laboratory evaluation, including CK measurement, and prompt initiation of supportive therapy are essential to prevent serious complications. Additionally, this report contributes to existing literature by expanding the recognized clinical spectrum of rhabdomyolysis associated with influenza A infection and emphasizes that extreme biochemical presentations can occur in healthy adults with favorable outcomes when managed appropriately.

## Conclusions

This case report highlights that rhabdomyolysis can occur as a complication of influenza A infection, even in young patients without comorbidities. Clinicians should consider this diagnosis in patients with flu-like symptoms and marked myalgia. Appropriate identification and management of rhabdomyolysis are essential to prevent serious complications such as acute kidney injury and avoid long-term sequelae. Further research is warranted to better understand the mechanisms and true incidence of rhabdomyolysis in influenza A infections. 
